# Limiting the Persistence of a Chromosome Break Diminishes Its Mutagenic Potential

**DOI:** 10.1371/journal.pgen.1000683

**Published:** 2009-10-16

**Authors:** Nicole Bennardo, Amanda Gunn, Anita Cheng, Paul Hasty, Jeremy M. Stark

**Affiliations:** 1 Department of Cancer Biology, Division of Radiation Biology, Beckman Research Institute of the City of Hope, Duarte, California, United States of America; 2 City of Hope Graduate School of Biological Sciences, Duarte, California, United States of America; 3 Department of Molecular Medicine/Institute of Biotechnology, The University of Texas Health Science Center at San Antonio, San Antonio, Texas, United States of America; The University of North Carolina at Chapel Hill, United States of America

## Abstract

To characterize the repair pathways of chromosome double-strand breaks (DSBs), one approach involves monitoring the repair of site-specific DSBs generated by rare-cutting endonucleases, such as I-SceI. Using this method, we first describe the roles of Ercc1, Msh2, Nbs1, Xrcc4, and Brca1 in a set of distinct repair events. Subsequently, we considered that the outcome of such assays could be influenced by the persistent nature of I-SceI-induced DSBs, in that end-joining (EJ) products that restore the I-SceI site are prone to repeated cutting. To address this aspect of repair, we modified I-SceI-induced DSBs by co-expressing I-SceI with a non-processive 3′ exonuclease, Trex2, which we predicted would cause partial degradation of I-SceI 3′ overhangs. We find that Trex2 expression facilitates the formation of I-SceI-resistant EJ products, which reduces the potential for repeated cutting by I-SceI and, hence, limits the persistence of I-SceI-induced DSBs. Using this approach, we find that Trex2 expression causes a significant reduction in the frequency of repair pathways that result in substantial deletion mutations: EJ between distal ends of two tandem DSBs, single-strand annealing, and alternative-NHEJ. In contrast, Trex2 expression does not inhibit homology-directed repair. These results indicate that limiting the persistence of a DSB causes a reduction in the frequency of repair pathways that lead to significant genetic loss. Furthermore, we find that individual genetic factors play distinct roles during repair of non-cohesive DSB ends that are generated via co-expression of I-SceI with Trex2.

## Author Summary

A deleterious lesion in DNA is a break of both strands, or a chromosome double-strand break (DSB). DSBs can arise during normal cellular metabolism, but are also a consequence of many forms of cancer therapy. If DSBs are not repaired prior to cell division, entire segments of a chromosome can be lost. Several pathways ensure that DSBs are repaired, though some pathways are prone to causing mutations and/or chromosomal rearrangements, each of which can contribute to cancer development. In the first part of this study, we describe the roles of individual genetic factors in distinct repair pathways of DSBs generated by the I-SceI endonuclease. From these studies, we find that some factors can function in multiple repair pathways. In the second part of this study, we present a method for partially degrading the cohesive DSB overhangs that are generated by I-SceI, which we find facilitates repair products that are not prone to being re-cut by the endonuclease. As a consequence, we have limited the persistence of such breaks, which we find causes a reduction in repair pathways that lead to significant genetic loss. As well, we use this method to characterize the role of individual genetic factors during the repair of non-cohesive DSB ends.

## Introduction

Chromosome double-strand breaks (DSBs) can be repaired by a number of mechanisms with a variety of mutagenic consequences [1]. In the context of ionizing radiation (IR) therapy or chemotherapy that utilizes DSB-inducing agents, such DNA damage in non-tumor cells could result in oncogenic mutations that cause secondary malignancies [2]. Thus, characterizing the factors and pathways that influence DSB repair will be important to develop therapeutic approaches that may limit the risk of secondary tumors, and to understand the etiology of genome rearrangements associated with primary cancer development.

DSB repair pathways show a varying propensity for genetic loss. A relatively precise form of repair is homology-directed repair (HDR) that uses the identical sister chromatid as a template for Rad51-mediated strand invasion and nascent DNA synthesis [1]. In contrast, end-joining (EJ) pathways are variably mutagenic, depending on the extent of end-processing and the fidelity of end-pairing. For instance, EJ via the V(D)J recombination nonhomologous end-joining (NHEJ) machinery has the potential to be precise, especially when DSB ends can be ligated without significant processing [3]. However, Ku-independent EJ (Alternative-NHEJ, Alt-NHEJ) often leads to deletion mutations, which are predominantly associated with short stretches of homology (microhomology) at repair junctions [4],[5]. Similar to Alt-NHEJ is single-strand annealing (SSA), which also causes deletions with homology at repair junctions, but involves extensive regions of homology [6]. In addition, for each of these pathways, loss of correct end-pairing during the repair of multiple simultaneous DSBs can lead to chromosomal rearrangements. For instance, EJ between distal ends of two tandem DSBs (Distal-EJ) results in loss of the chromosomal segment between the DSBs.

To characterize the genetic factors that influence these pathways, one approach involves analyzing repair of site-specific DSBs in mammalian cells, such as those generated by the rare-cutting endonuclease I-SceI. For instance, using this approach, HDR, SSA, and Alt-NHEJ were shown to be promoted by CtIP and Nbs1 [7]–[10], which are factors implicated in the formation of ssDNA via end resection [9],[11]. As well, the strand exchange factors Rad51/Brca2 were found to promote HDR and suppress SSA [12],[13], and a number of additional genetic factors have been found to promote HDR [14]. Other studies have addressed the influence of factors involved in NHEJ during V(D)J recombination, including Ku and Xrcc4-Ligase IV. For example, Ku/Xrcc4-deficient cells show higher HDR [15], and Ku-deficient cells show elevated SSA and Alt-NHEJ [5]. In addition, Ku and Xrcc4 have been shown to promote EJ that restores the I-SceI site, measured as EJ between distal ends of two tandem I-SceI-induced DSBs (S+DEJ) [16],[17].

To further address the process of DSB repair pathway choice in mammalian cells, we have developed this two-part study. In the first part, we provide a detailed characterization of the roles of Ercc1, Msh2, Nbs1, Xrcc4, and Brca1 during individual repair events. From these studies, we provide evidence that individual genetic factors may not be specific for particular pathways of repair, but rather promote a mechanistic step that is common among distinct repair pathways. Regarding particularly distinct findings, we present evidence that Msh2 promotes HDR, whereas Ercc1 is particularly required for repair events that require removal of a nonhomologous segment. Moreover, these experiments provide essential reagents for the development of the second part.

In the second part of this study, we have addressed whether the outcome of these repair assays could be affected by the persistent nature of I-SceI-induced DSBs. Namely, since precise EJ restores the I-SceI site, chromosomal I-SceI sites are prone to repeated cutting by the I-SceI endonuclease, which has been referred to as the persistent nature of endonuclease-generated DSBs [18]–[21]. To address this aspect of repair, we expressed a 3′ exonuclease, Trex2 [22],[23], to partially degrade the 3′ overhangs generated by I-SceI, and thereby promote EJ products that have lost the I-SceI site. Since these EJ products are resistant to further cutting by I-SceI, we suggest that Trex2 expression can limit the persistence of I-SceI-induced DSBs.

Using this approach, we find that Trex2 expression strongly decreases the frequency of Distal-EJ in favor of EJ events that maintain proximal end-pairing. Trex2 expression also causes a significant decrease in Alt-NHEJ and SSA. In contrast, HDR is not inhibited by Trex2 expression. These results indicate that limiting the persistence of DSBs can suppress repair pathways that are prone to genetic loss. As well, using this Trex2 approach, we find that individual genetic factors play distinct roles during repair of non-cohesive DSB ends.

## Results

### Reporters for distinct DSB repair events

To investigate the genetic requirements of individual DSB repair pathways, as well as the effect of the persistence of a DSB on repair, we have developed a series of reporters for discrete repair events. In each case, we generate an I-SceI-induced DSB within a chromosomally integrated inactive *GFP* cassette, where the structure of each reporter is designed such that repair of the DSB by a specific pathway results in restoration of the *GFP+* cassette. For instance, three reporters were designed to measure distinct end-joining (EJ) events, as described previously [7], and summarized below.

**Figure 1:**
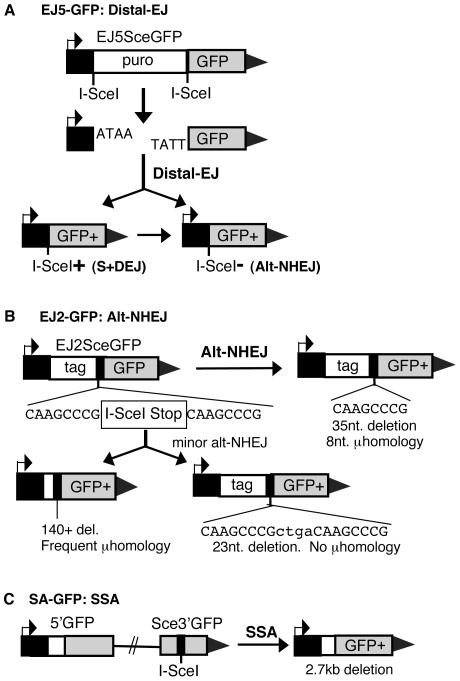
Reporters for EJ and SSA repair pathways. (A) EJ5-GFP is shown along with products of EJ between distal DSB ends (Distal-EJ) that restores a GFP expression cassette, including Ku-dependent I-SceI site restoration (S+DEJ), and Ku-independent I-SceI-resistant products (Alt-NHEJ). (B) EJ2-GFP is shown along with products of Alt-NHEJ that restore the reading frame of the GFP expression cassette. The most abundant Alt-NHEJ product involves 8 nt of microhomology at the repair junction and a 35 nt deletion. (C) SA-GFP is shown along with the SSA repair product that utilizes 266 nt of homology between the tandem GFP segments, thereby restoring a GFP expression cassette.

First, the EJ5-GFP reporter measures end-joining between distal ends of two tandem I-SceI-induced DSBs (Figure 1A [7]). This Distal-EJ product results in loss of a fragment between the two I-SceI sites (*puro* gene), and thereby restores the juxtaposition of the promoter next to the remainder of the *GFP* cassette. This repair product was previously referred to as total-NHEJ, but Distal-EJ is a more precise description of these repair events, since proximal-EJ would lead to maintenance of the fragment between the two I-SceI sites, and not lead to a *GFP+* cassette. Such Distal-EJ can result in either reconstitution of the I-SceI site (S+DEJ) or generation of an I-SceI-resistant site. In previous work with this reporter, Ku70 was shown to be essential for S+DEJ events, but completely dispensable for I-SceI-resistant EJ events [7]. As well, the repair junctions of I-SceI-resistant EJ events were shown to predominantly exhibit microhomology (90%) [7]. The findings that I-SceI-resistant EJ products are elevated in Ku-deficient cells, and show evidence of microhomology, suggest that these events are one measure of Alt-NHEJ. However, Ku70 may play an important role during a subclass of I-SceI-resistant EJ events that involve minimal microhomology [16].

Another reporter, EJ2-GFP, specifically measures such Alt-NHEJ events (Figure 1B, [7]). This reporter involves a single I-SceI-induced DSB within a disrupted *GFP* coding sequence, where a discrete set of Alt-NHEJ events restores a functional *GFP* cassette. The predominant *GFP+* product utilizes 8 nucleotides (nt) of microhomology that flank the DSB, which results in a 35 nt deletion. Other Alt-NHEJ events with different deletion sizes can also restore the *GFP+* cassette, though these products are less frequent (15% of total products). Importantly, the GFP+ repair events measured with EJ2-GFP have been shown to be suppressed by Ku70 [7], which further indicates that this reporter measures Alt-NHEJ.

Finally, the SA-GFP reporter measures SSA between two *GFP* fragments that share 266 nt of homology and are separated by 2.7 kb, where an I-SceI site is present in the downstream *GFP* fragment (Figure 1C). Notably, while this SSA event involves a significant stretch of homology, such repair is suppressed by the homologous strand-exchange factor RAD51 [8]. This finding that the GFP+ product from SA-GFP is suppressed by RAD51, combined with the relatively low frequency of HDR associated with crossing-over and/or long gene conversion tracts [24],[25], suggests that such rare HDR events do not likely contribute significantly to the formation of the GFP+ product in SA-GFP [8].

### The reporter DRins-GFP provides a bridge between HDR and SSA

**Figure 2:**
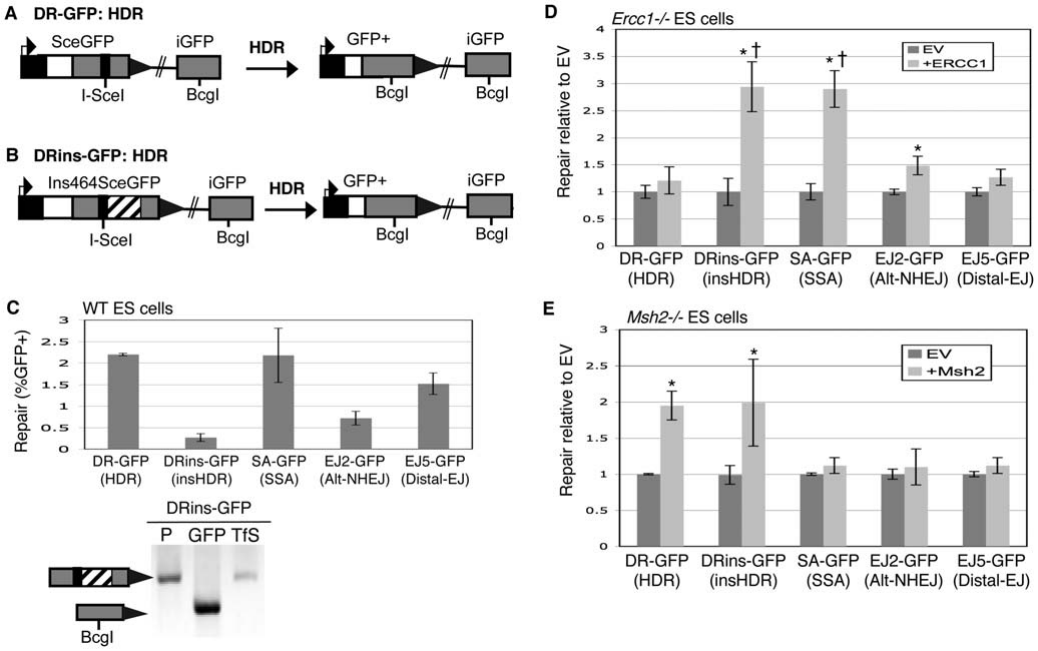
Ercc1 promotes repair pathways that require removal of a nonhomologous segment, whereas Msh2 promotes HDR. (A) DR-GFP is shown along with the HDR product that uses *iGFP* as the template for nascent DNA synthesis, which results in restoration of a GFP expression cassette. (B) Shown is a distinct reporter for HDR (DRins-GFP) that is similar to DR-GFP, except it contains an insertion of 464 nt downstream of the I-SceI site that needs to be removed during HDR to restore a GFP expression cassette. (C) Requiring removal of a nonhomologous insertion during HDR decreases the efficiency of repair. DR-GFP and DRins-GFP were integrated at the identical locus (*Pim1*) of wild-type (J1 strain) mouse ES cells, and the efficiency of HDR (%GFP+ cells) was determined following transient expression of I-SceI. Shown is the efficiency of repair (%GFP+ cells) from these reporters, along with those from Figure 1 that are also in WT ES cells. Also shown is confirmation of the structure of the *GFP+* repair product for DRins-GFP via PCR/sequencing analysis of sorted GFP+ cells. The labels P, GFP, and TfS denote the parental cell line, GFP+ sorted cells, and total I-SceI-transfected cells, respectively. (D) Ercc1 specifically promotes DSB repair pathways that require removal of an extended nonhomologous segment. Each of the reporters depicted in Figure 1 and Figure 2A and 2B were integrated into mouse ES cells deficient in Ercc1. These individual cell lines were transfected with an expression vector for I-SceI, along with either a complementation vector for Ercc1, or the empty expression vector (EV). Repair is measured as percent GFP+ cells, which is normalized to the EV samples transfected in parallel. Asterisks denote a statistical difference in repair efficiency from EV (p<0.0001). The dagger denotes a statistical difference in fold-complementation compared to HDR of the DR-GFP reporter (p<0.0001). (E) Msh2 specifically promotes HDR. Analysis of repair in *Msh2−/−* cells was performed as described above for Ercc1, where asterisks denote the same statistical differences as described above.

Distinct from the above reporters for EJ, the DR-GFP reporter is designed to measure HDR (Figure 2A), where a gene fragment (*iGFP*) serves as a template for RAD51-mediated HDR of an I-SceI-induced DSB in an upstream *SceGFP* cassette [12]. However, these *GFP* segments differ by only 11 point mutations (see Figure S1A [26]); therefore, DR-GFP does not require removal of a large segment during repair. In contrast, HDR of complex lesions, such as a series of inter-strand crosslinks (ICLs), and HDR between divergent sequences, could require removal of a significant chromosomal segment to complete repair [27].

To begin addressing this aspect of HDR, we developed another reporter, DRins-GFP (Figure 2B), which is designed to require removal of a nonhomologous segment during HDR. Specifically, this reporter contains a 464 nt insertion of mouse genomic sequence (intron segment of the *Rb* gene) placed downstream of the I-SceI site in *SceGFP* (*Ins464SceGFP*). Removal of this insertion would be critical for resolution of the HDR product, but also may be important to disrupt attempts to strand invade the insertion sequence at the *Rb* locus. To analyze this reporter, both DR-GFP and DRins-GFP were integrated into the *pim1* locus of wild-type (WT) mouse ES cells. We used ES cells for this study because of the prevalence of specific mutant cell lines, but also because of the relevance of stem cells in regenerative medicine and the etiology of cancer [28]. Subsequently, we transfected these cell lines with an expression vector for I-SceI, and determined the efficiency of HDR by FACS analysis of GFP+ cells. For completion, we also included cell lines with the reporters in Figure 1 in these experiments, and confirmed the structure of the GFP+ product for DRins-GFP (Figure 2C). Regarding a direct comparison between the HDR reporters, we found that HDR of the DRins-GFP reporter was significantly less efficient than for DR-GFP (8-fold, p<0.0001, Figure 2C). This result indicates that HDR is impaired by the insertion, which also suggests that HDR repair of the DRins-GFP reporter may have unique mechanistic requirements relative to HDR of DR-GFP. Though, as an alternative interpretation, attempts to strand invade the insertion at the *Rb* locus could contribute to the low efficiency of HDR of the DRins-GFP reporter.

Regarding the possibility of distinct mechanistic requirements between these HDR events, we considered the notion that HDR repair of the DRins-GFP reporter may share a common mechanistic step with SSA, thereby providing a bridge between HDR and SSA. Namely, HDR of DRins-GFP is similar to SSA repair of SA-GFP in that both require removal of an extended segment, whereas HDR of DR-GFP does not. So, we hypothesized that HDR repair of DRins-GFP may share end-processing steps with SSA repair of SA-GFP. Such a processing step could involve extensive 5′ to 3′ resection, and/or cleavage of the insertion as an unpaired 3′ ssDNA tail. In particular, we addressed the hypothesis that 3′ ssDNA tail removal, via Ercc1, may be a common step between SSA and HDR of the DRins-GFP reporter. Ercc1 forms a complex with Xpf and is involved in endonucleolytic cleavage of 3′ ssDNA [29], which supports a role for Ercc1 during processing of 3′ ssDNA. Furthermore, Ercc1 has been shown to promote SSA [8], as well as EJ deletion products during joining of plasmid substrates [30].

To test the above hypothesis, we integrated DRins-GFP into an Ercc1-deficient mouse ES cell line (*Ercc1−/−*), in which both alleles of *Ercc1* were targeted with selection cassettes near the 3′ end of the gene [31]. Then, we transfected this cell line with an expression vector for I-SceI, along with either a complementation vector for Ercc1, or the associated empty vector (EV). As well, we performed this set of transfections on a set of *Ercc1−/−* cell lines with each of the other reporters in Figure 1 and Figure 2, many of which have been described previously [7]. Expression of Ercc1 via the complementing vector was confirmed by immunoblotting (Figure S1B). Subsequently, we quantified the fold-effect of the complementation vector on the efficiency of repair, as compared to parallel transfections with EV.

We have found that quantifying such fold-complementation provides the most consistent means for determining the influence of a given genetic factor. Importantly, we have not observed any clear effects on viability or proliferation resulting from complementation in any of the genetic analysis in this study (unpublished observations). In any case, such variations are rare in mouse ES cells, given their high rate of proliferation, lack of a p53-dependent G1/S checkpoint, and short gap phases (G1/G2) [32]. As an alternative, we have included the overall frequency of repair for each of the below experiments, to allow for a direct comparison across different cell lines, which yields the same basic conclusions as the complementation experiments (Figure S3).

From these experiments (Figure 2D), we found that Ercc1 complementation showed a significant increase in the efficiency of HDR of the DRins-GFP reporter (2.9-fold), and showed the same effect on SSA (2.9-fold). In contrast, consistent with previous results [7], Ercc1 played a minor role in Alt-NHEJ (EJ2-GFP, 1.5-fold), and insignificant roles in HDR of the DR-GFP reporter and Distal-EJ (DR-GFP, 1.2-fold; EJ5-GFP, 1.3-fold). These results indicate that Ercc1 is particularly important for DSB repair involving processing of long nonhomologous segments, rather than SSA per se.

The above analysis with Ercc1 provides an example of how a genetic factor may not be specific for an individual repair pathway, but rather promotes a mechanistic step that may arise during multiple repair events. To provide further evidence for this notion, we next present a similar analysis with other genetics factors. In addition, we will be including many of the reagents from this genetic analysis during our later description of experiments involving expression of Trex2.

### Msh2 promotes HDR

Since Msh2 is important for the mechanistic step of mismatch detection during mismatch repair [33], we wondered whether this factor might also be important for other pathways of repair in mammalian cells. We analyzed the five reporters described in Figure 1 and Figure 2 using *Msh2−/−* ES cells [34], and the complementation approach described for Ercc1. Notably, expression of Msh2 from the complementing vector was confirmed by immunoblotting (Figure S1B). From these experiments (Figure 2E), we found that Msh2-complementation promotes HDR of both the DR-GFP and DRins-GFP reporters (2-fold). In contrast, we found that Msh2-complementation had no effect on the overall efficiency of Alt-NHEJ, or Distal-EJ, which is consistent with previous studies in hamster (CHO) cells [35]. Furthermore, we find that Msh2-complementation has no clear effect on SSA, which is distinct from the role of Ercc1 in mammalian cells shown above, and the role of *MSH2* during SSA in yeast [36]–[38]. This distinction between Ercc1 and Msh2 during HDR is further developed in experiments with Trex2, in that Trex2 expression promotes HDR in *Msh2−/−* but not *Ercc1−/−* cells (see below). In summary, we find that Msh2 is specifically important for HDR, and shows distinct roles during DSB repair compared to Ercc1.

### Nbs1 promotes repair that requires some degree of homology, but is dispensable for S+DEJ

We continued with an analysis of the role of Nbs1 during repair. Previously, an Mre11-complex (Mre11-Rad50-Nbs1) interacting factor, CtIP [9],[11], was shown to promote HDR, SSA, and Alt-NHEJ, but was found to be dispensable for Distal-EJ [7]. As well, Nbs1 and Mre11 have recently shown to promote Alt-NHEJ [10], [39]–[41]. We sought to further investigate the role of Nbs1 during EJ, perform a comparative analysis of the role of Nbs1 during multiple pathways of repair, and develop reagents used in the below Trex2 experiments.

For this analysis, we used a double-targeted *Nbs1^n/h^* mouse ES cell line that was generated in a previous study, in which the *Nbs1* gene was targeted at both alleles by *neo (n)* and *hyg (h)* cassettes, such that these cells were previously shown to lack any Nbs1 protein [42]. However, this result contradicts the notion that the MRE11-complex appears essential for viability of mouse ES cells [43]. Also, while the targeting constructs were designed to remove exon 6, only one such double-targeted clone was isolated [42], raising the possibility that one allele may involve an aberrant targeting event that merely causes a decrease in Nbs1 expression, similar to an ES cell line deficient in the Blm helicase [44].

**Figure 3:**
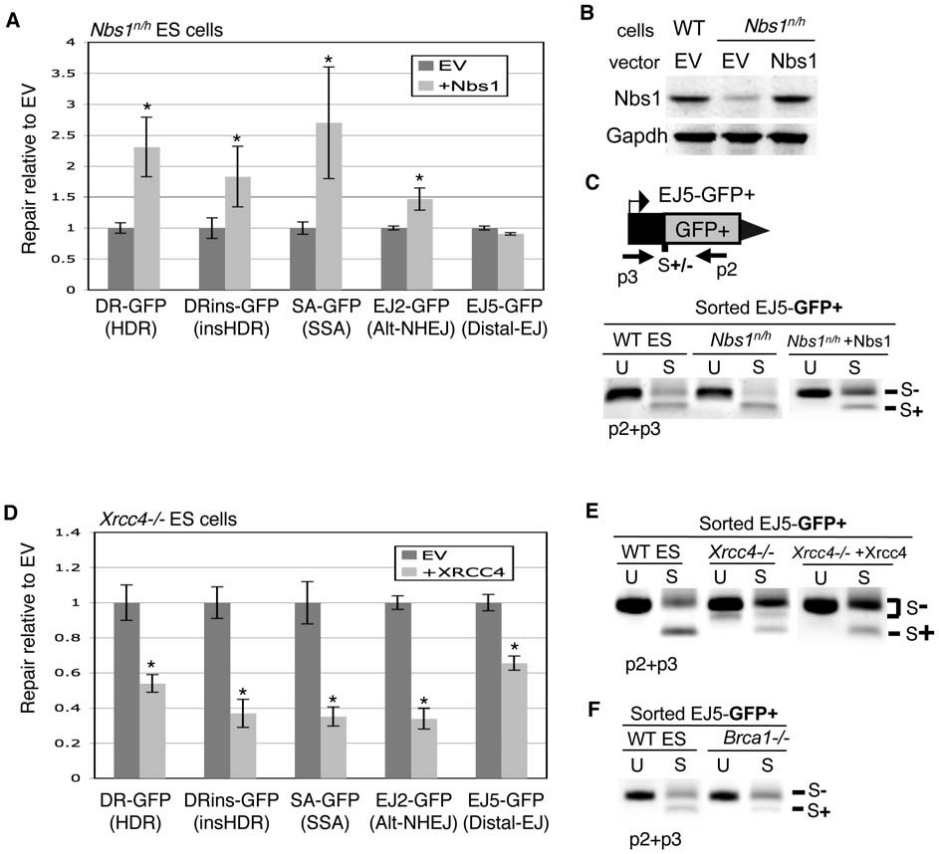
Nbs1, Xrcc4, and Brca1 play distinct roles during individual repair events. (A) Nbs1 promotes HDR, SSA, and Alt-NHEJ, but is dispensable for Distal-EJ. Reporters from Figure 1 and Figure 2 were integrated into *Nbs1^n/h^* cells, and the effect of Nbs1 complementation on repair was determined as for Ercc1 in Figure 2D. Asterisks denote a statistical difference in repair efficiency from EV (p<0.0001). (B) *Nbs1^n/h^* cells show a reduced level of Nbs1 that is restored to wild-type levels with transient expression. Shown are immunoblot signals for Nbs1 from transfections with *Nbs1^n/h^*, and from WT cells. (C) The repair event S+DEJ is increased in Nbs1-deficient cells. The diagram depicts the primers used for amplification (p2, p3). Shown are amplification products from sorted GFP+ cells derived from I-SceI transfection of EJ5-GFP-containing WT and *Nbs1^n/h^* cells, as well as *Nbs1^n/h^* cells transiently complemented with Nbs1. The products have been left uncut (U) and cut with I-SceI (S). (D) Xrcc4 suppresses HDR, SSA, Alt-NHEJ, and Distal-EJ. Shown are repair levels of reporters integrated into *Xrcc4−/−* ES cells that were assayed with/without transient complementation of Xrcc4 as described for Ercc1 in Figure 2D. Asterisks denote a statistical difference in repair efficiency from EV (p<0.0001). (E) Xrcc4-deficient cells show a decrease in S+DEJ. Shown are amplification products using the same primers and annotation as shown in C, from sorted GFP+ cells derived from I-SceI transfection of EJ5-GFP-containing WT cells, *Xrcc4−/−* cells, and *Xrcc4−/−* cells transiently complemented with Xrcc4 as in D. (F) Brca1-deficient cells show a decrease in S+DEJ. As in E, amplification products are shown from sorted GFP+ cells derived from I-SceI transfection of EJ5-GFP for WT and *Brca1−/−* cell lines.

Accordingly, we tested whether the *Nbs1^n/h^* cell line still expresses intact full-length Nbs1, but at a substantially lower level. For this, we performed immunoblot analysis using an anti-Nbs1 antibody on whole cell extracts from *Nbs1^n/h^* cells, and found an immunoblot signal at the correct size for Nbs1 that co-migrated with the Nbs1 signal in WT (see Figure 3B). Importantly, the Nbs1 immunoblot signal in *Nbs1^n/h^* cells was substantially lower than WT (at least 5-fold reduction, see Figure 3B). The difference between this analysis and the previous study showing no Nbs1 immunoblot signal, using the identical cell line [42], may reflect variations in the sensitivity of immunoblotting. Nevertheless, the *Nbs1^n/h^* cell line is clearly deficient in wild-type levels of Nbs1, which can be complemented with transient expression of Nbs1 (see Figure 3B).

*Nbs1^n/h^* cells were previously shown to exhibit reduced HDR and SSA [42], where Alt-NHEJ was not directly addressed in this study. To test the role of Nbs1 during multiple pathways, we generated *Nbs1^n/h^* mouse ES cell lines with an integrated copy of each reporter in Figure 1 and Figure 2B. The parental *Nbs1^n/h^* cells and the DR-GFP *Nbs1^n/h^* cell line were obtained directly from the laboratory that generated these reagents [42]. Using these cell lines, we evaluated the fold-effect of complementation of Nbs1 on repair in the *Nbs1^n/h^* cells, using the same approach as described for Ercc1. From these experiments (Figure 3A), we found that HDR and SSA were both promoted by Nbs1-complementation (DR-GFP, 2.3-fold; DRins-GFP, 1.8-fold; and SA-GFP, 2.7-fold), consistent with the previous study with these cells [42]. As well, from comparison of SA-GFP repair frequencies between *Nbs1^n/h^* and WT cells, the role of Nbs1 during SSA is even more pronounced (Figure S3C).

With respect to the EJ reporters in the *Nbs1^n/h^* cells (Figure 3A), we found that Alt-NHEJ (EJ2-GFP) was promoted by Nbs1 complementation (1.5-fold), whereas Distal-EJ (EJ5-GFP) was unaffected. For another measure of Alt-NHEJ, using EJ5-GFP, we quantified the relative ratio of I-SceI-restoration (S+DEJ) to I-SceI-resistant EJ products during Distal-EJ (see Figure 1A). With this analysis, a defect in Alt-NHEJ would be expected to cause an increase in the proportion of S+DEJ events. To quantify this repair event, we amplified a region surrounding the I-SceI site in the EJ5-GFP reporter using sorted GFP+ cells, followed by I-SceI digestion analysis. During such analysis, we ensure that all our experiments are performed under conditions for complete I-SceI digestion [7],[15], which includes limiting the amount of amplification product [45], as well as performing digestion analysis of control amplification products with an intact I-SceI site (see Materials and Methods). Using this method, we found that S+DEJ events are increased in *Nbs1^n/h^* cells relative to WT (1.6+/−0.1-fold, p<0.0001, Figure 3C, Figure S1C). We also found that transient complementation of Nbs1 in *Nbs1^n/h^* cells reduced S+DEJ products back to near WT levels (Figure 3C, Figure S1C). Thus, Nbs1 appears to promote Alt-NHEJ, but is dispensable for S+DEJ. Thus, we suggest that Nbs1 is important for a number of repair events that require access to homology.

### Xrcc4 promotes S+DEJ, and suppresses Alt-NHEJ, SSA, HDR, and the total frequency of Distal-EJ

We next addressed the role of Xrcc4 during repair, which is a factor that binds to Ligase IV and promotes both its stability and function during NHEJ [46]. In previous studies, *Xrcc4−/−* mouse ES cells have been shown to exhibit elevated levels of HDR [15]. We extended the analysis of these *Xrcc4−/−* ES cells [47], using the reporters and complementation approach described above, where expression of Xrcc4 from the complementing vector was confirmed by immunoblotting (Figure S1B). In particular, we performed these experiments to address the role of Xrcc4 during SSA, and to establish reagents used for the below analysis of EJ using Trex2.

From these experiments (Figure 3D), we found that Xrcc4 complementation resulted in a significant inhibition of HDR (DR-GFP, 1.8-fold; DRins-GFP, 2.7-fold), SSA (SA-GFP, 2.8-fold), Alt-NHEJ (EJ2-GFP, 2.9-fold), and Distal-EJ (EJ5-GFP, 1.5-fold). To characterize the nature of EJ events in Xrcc4-deficient cells, we determined the efficiency of I-SceI-restoration (S+DEJ) during Distal-EJ, using amplification analysis of GFP+ sorted cells from the EJ5-GFP transfections, as described above for Nbs1. From this analysis, we found that the efficiency of S+DEJ is reduced in *Xrcc4−/−* ES cells relative to WT cells (2.8+/−0.2-fold, p<0.0001, Figure 3E, Figure S1C). As well, the *Xrcc4−/−* cells show an additional class of smaller I-SceI-resistant products, indicative of extensive deletions during EJ (Figure 3E). Next, we performed this EJ analysis on GFP+ sorted cells following co-expression of I-SceI with Xrcc4 in *Xrcc4−/−* cells. From this experiment, we found that Xrcc4 expression suppressed the formation of extensive deletion products, suggesting that transient complementation of Xrcc4 can restore its end-protection functions. In contrast, co-expression of I-SceI and Xrcc4 caused only a partial restoration of the efficiency of S+DEJ in *Xrcc4−/−* cells (1.5-fold increase relative to *Xrcc4−/−*, p = 0.0008, Figure 3E, Figure S1C). This result may reflect an inability to completely restore the ligase functions of Xrcc4-Ligase IV by transient complementation. However, even comparing *Xrcc4−/−* versus WT for the efficiency of S+DEJ, we find that Xrcc4 is not absolutely required for this repair event. Thus, other ligase complexes may be able to complete the S+DEJ event, particularly since this product could be stabilized by the microhomology of the cohesive I-SceI overhangs [46].

In summary, these data indicate that Xrcc4 plays some role in S+DEJ events, and suppresses SSA, Alt-NHEJ, HDR, and Distal-EJ. We suggest that suppression of HDR, SSA, and Alt-NHEJ could result from the end-protection function of Xrcc4 [48], which may limit end resection during these pathways. In contrast, the finding that Xrcc4-complementation suppresses Distal-EJ may reflect a role for Xrcc4 is supporting EJ between proximal ends.

### Brca1 promotes S+DEJ and inhibits the total frequency of Distal-EJ

For comparison with Nbs1 and Xrcc4, we also determined the effect of Brca1-deficiency on repair of the EJ5-GFP reporter. Also, we introduce this cell line here, as it is used below for additional EJ experiments with Trex2 (see below). Specifically, we integrated EJ5-GFP into mouse ES cells that are homozygous for an exon 11-deletion allele (*Brca1−/−*), which encodes a protein with a substantial internal deletion [49],[50]. The size of Brca1 has made transient complementation unfeasible, such that we have been limited to a comparison of repair versus WT. The reporters DR-GFP and SA-GFP have already been analyzed in this *Brca1−/−* cell line, showing a 5.3-fold and a 1.8-fold decrease relative to WT ES cells, in HDR and SSA, respectively [8]. Using the *Brca1−/−* EJ5-GFP cell line, we expressed I-SceI and subsequently determined the frequency of Distal-EJ. As well, we quantified the relative efficiency of S+DEJ versus I-SceI-resistant Distal-EJ products in GFP+ sorted cells. From these experiments, we found that the total frequency of Distal-EJ (%GFP+) was increased in *Brca1−/−* versus WT ES cells (2-fold, p<0.0001, Figure S3E). As well, from quantification of S+DEJ from GFP+ cells, we found a significant decrease in this repair event in *Brca1−/−* cells compared to WT ES cells (3-fold decrease, Figure 3F, Figure S1C). Thus, Brca1 promotes S+DEJ, which may indicate that Brca1 is important for EJ of cohesive ends. Based on this notion, Brca1 could feasibly promote S+EJ at proximal ends, which may account for the suppression of Distal-EJ. While these proximal S+EJ events cannot be quantified, this model is supported by other reports showing a role for Brca1 during EJ of plasmid substrates with cohesive ends [51],[52], and are developed with the below Trex2 experiments.

### Expression of Trex2 promotes formation of I-SceI-resistant EJ products that are dependent on Xrcc4

We next considered the possibility that the outcome of these studies on repair may be affected by the unstable nature of EJ products that restore the I-SceI site, which are prone to repeated cutting by I-SceI. This property of endonuclease-generated DSBs has been referred to as the persistent nature of such DSBs in previous studies [18]–[21]. Thus, we developed a method to promote the formation of I-SceI-resistant EJ products, and thereby limit the persistent nature of I-SceI-induced DSBs. We then used this approach to address how the relative persistence of DSBs may affect the mutagenic consequences of such damage. For this, we co-expressed I-SceI with a protein that we predicted would catalyze partial degradation of the 3′ ssDNA 4 nt overhangs generated by I-SceI, and hence promote formation of EJ products that are resistant to cleavage by I-SceI. Specifically, we expressed mammalian Trex2, which is a potent non-processive 3′ to 5′ exonuclease [22],[23],[53].

**Figure 4:**
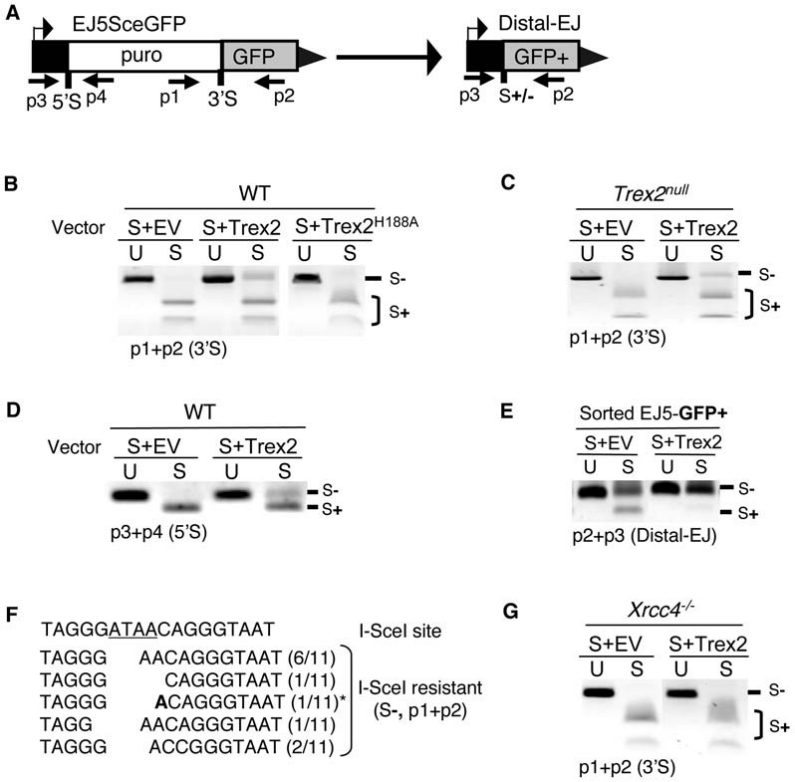
Expression of the Trex2 exonuclease promotes the formation of Xrcc4-dependent I-SceI-resistant EJ products. (A) Shown are primers for EJ5-GFP that are used to analyze proximal-EJ at the 5′ and 3′ I-SceI sites (shown as 5′S and 3′S, respectively), as well as Distal-EJ. (B) Expression of the Trex2 exonuclease promotes the formation of I-SceI-resistant EJ products between proximal DSB ends at the 3′ I-SceI site in EJ5-GFP. WT ES cells with EJ5-GFP were transfected with an I-SceI expression vector (S) along with a Trex2 expression vector (Trex2), an exonuclease-deficient mutant of Trex2 (Trex2-H188A), or EV. Shown are amplification products from these transfections using the primers p1 and p2, which were either left uncut (U) or were cut with I-SceI (S). (C) Trex2 expression also promotes I-SceI-resistant EJ products in *Trex2^null^* cells. Analysis was performed on *Trex2^null^* cells with the EJ5-GFP reporter as described in B. (D) Trex2 expression promotes I-SceI-resistant EJ products at the 5′ I-SceI site of EJ5-GFP. Shown are amplification products using the primers p3 and p4, using the same transfection conditions and annotation as described in B. (E) Trex2 expression promotes I-SceI-resistant Distal-EJ products. Shown are amplification products from sorted GFP+ cells derived from the transfections shown in C, using primers p3 and p2, with the same annotation shown in B. (F) EJ products via Trex2 show deletion of segments of the I-SceI 3′ overhang (underlined). The I-SceI-resistant products shown in C were cloned, and 11 individual clones were sequenced. Shown are the sequences of these clones, where the numerator in parenthesis depicts the number of times a given sequence was identified. An asterisk denotes the one clone with evidence of microhomology (1 nt., A in bold). (G) I-SceI-resistant proximal EJ products via Trex2 are dependent on Xrcc4. Analysis of the effect on Trex2 expression on the 3′ I-SceI site of the EJ5-GFP reporter was performed on *Xrcc4−/−* cells as described in B.

We first determined whether Trex2 expression promotes EJ products that are resistant to cleavage by I-SceI, using the EJ5-GFP reporter. In these experiments, transfection of the Trex2 expression vector leads to at least a 10-fold increase of Trex2 mRNA above WT, largely due to the relatively low endogenous level of Trex2 expression in these cells, based on quantitative RT-PCR (data not shown). Following transfection of I-SceI along with Trex2 or EV, we quantified the formation of I-SceI-resistant EJ products. Regarding this analysis of two tandem I-SceI induced DSBs, three different sets of ends can be paired during EJ. Two of these end-pairs result in retention of the intervening *puro* cassette: pairing of the proximal ends that flank the 3′ I-SceI site, and pairing of the proximal ends that flank the 5′ I-SceI site. In contrast, pairing of the distal ends of the 5′ and 3′ I-SceI sites (Distal-EJ) results in loss of the intervening *puro* gene. To quantify formation of I-SceI-resistant EJ products for each of these end-pairs, we amplified the region surrounding each EJ event (Figure 4A), and subjected the amplification products to I-SceI digestion analysis.

From this analysis, we found that Trex2 expression results in the formation of I-SceI-resistant EJ products between proximal ends of the 3′ I-SceI site (24%+/−8% and 27%+/−5% of total amplified product in WT and *Trex2^null^* ES cells [22], respectively (Figure 4B and 4C). In addition, we found a similar effect of Trex2 expression on EJ between proximal ends of the 5′ I-SceI site (30%+/−7 I-SceI-resistant products in WT ES cells, Figure 4D). In contrast, in the absence of Trex2 expression, these I-SceI-resistant proximal-EJ products were not detectable (see S+EV, Figure 4B–4D). Regarding Distal-EJ, Trex2 expression caused a substantial increase in the fraction of I-SceI-resistant products, in that S+DEJ products were undetectable in the GFP+ repair events from cells transfected with Trex2 (Figure 4E). Thus, Trex2 expression promotes the formation of I-SceI-resistant EJ products in EJ5-GFP, between proximal ends at both the 5′ and 3′ I-SceI sites, as well as during Distal-EJ.

We next addressed whether the exonuclease activity of Trex2 is involved in its ability to promote I-SceI-resistant EJ products. To begin with, we characterized the repair junctions of the Trex2-mediated I-SceI-resistant EJ products at the 3′ I-SceI site, by cloning these products and sequencing individual clones. From this analysis, we found sequences that are consistent with exonucleolytic processing of the 3′ overhangs (Figure 4F). For example, the most abundant product (6/11, 54%) shows mutation of the 3′ overhang ATAA/TATT to AA/TT. Notably, only one product (1/11, 9%) showed any evidence of microhomology (1 nt. microhomology, ATAA/TATT to A/T). Thus, the structures of these EJ products are consistent with the known non-processive 3′ to 5′ exonuclease activity of Trex2 [22],[23],[53].

In addition, we characterized a mutant form of Trex2 (H188A), which has been shown to lack exonuclease activity, but retains significant DNA binding activity (reduced only 60% from Trex2-WT) [54]. For this, we co-transfected expression vectors for Trex2-H188A and I-SceI into WT ES cells with EJ5-GFP, using identical conditions as the previous experiments with wild-type Trex2. From these experiments, we found that the Trex2-H188A mutant caused no detectable formation of I-SceI-resistant EJ products at the 3′ I-SceI site (see Figure 4B). Along these lines, we also wanted to address whether Trex2 expression caused an overall increase in DNA damage, as assessed by immunoblotting of a marker for chromosome breaks, γH2AX [55]. We found that transfection of Trex2 had no affect on the level of γH2AX, as compared to spontaneous γH2AX levels from parallel EV transfections (Figure S2A), which is consistent with previous reports showing expression of wild-type Trex2 does not cause an increase in chromosome breaks [53].

Given that Trex2-mediated EJ products do not involve substantial amounts of microhomology (see Figure 4F), we hypothesized that these repair events might be dependent upon Xrcc4, since Xrcc4-Ligase IV is particularly effective at ligating substrates that are not stabilized by annealing [56]. To test this, we co-transfected the Trex2 and I-SceI expression vectors in *Xrcc4−/−* cells with the EJ5-GFP reporter. We then quantified the formation of I-SceI-resistant EJ products at the 3′ I-SceI site, as described for WT cells (see Figure 4B). From these experiments, we reproducibly found no detectable level of I-SceI-resistant proximal EJ products from Trex2 expression in *Xrcc4−/−* cells (Figure 4G), where such products were readily detected in WT cells (see Figure 4B). This result indicates that EJ of ends processed by Trex2 is dependent upon Xrcc4, which may reflect a critical role for Xrcc4-Ligase IV during ligation of ends that do not contain substantial microhomology. Consistent with this notion, Xrcc4 is much less important for I-SceI-restoration (see Figure 3E), while the 4 nt of microhomology from the I-SceI overhangs might allow EJ by other ligase complexes [46].

In total, these data support the notion that the exonuclease activity of Trex2 catalyzes partial degradation of I-SceI DSB overhangs, thereby promoting the formation of I-SceI-resistant EJ products. However, it is certainly possible that Trex2 additionally could be recruiting other factors to facilitate the EJ process. In any case, co-expression of Trex2 and I-SceI appears to result in I-SceI-resistant EJ products. Since these products cannot be repeatedly cut by I-SceI, we suggest that Trex2 expression can limit the persistent nature of I-SceI-induced DSBs.

### Limiting the persistence of DSBs via Trex2 reduces the frequency of Distal-EJ, SSA, and Alt-NHEJ, but not HDR

**Figure 5:**
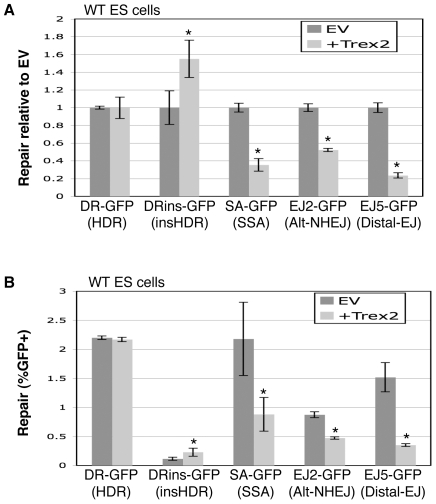
Trex2 expression causes a significant decrease in Distal-EJ, Alt-NHEJ, and SSA, but not HDR. WT ES cell lines with individual reporters were co-transfected with expression vectors for I-SceI and Trex2. (A) Shown are repair values normalized to parallel co-transfections with I-SceI and EV. Asterisks denote a statistical difference from EV (p<0.0001, DRins-GFP p = 0.0008). (B) Shown are primary repair values (%GFP+) from the experiment shown in A, to allow a direct comparison of the frequencies of different repair events. The error bars are somewhat larger in this panel as compared to A, since the primary repair levels show greater experimental variation versus the relatively consistent fold-effect of Trex2 expression. Asterisks denote a statistical difference from EV (p<0.0001 for EJ2-GFP and EJ5-GFP, p = 0.001 for SA-GFP, and p = 0.005 for DRins-GFP).

We then considered whether expression of Trex2 affects the relative efficiency of distinct repair events, beginning with the reporters described in Figure 1. From these experiments (Figure 5A and 5B), we found that co-expression of Trex2 with I-SceI in WT ES cells caused a striking decrease in the efficiency of Distal-EJ (4.2-fold), as well as a significant decrease in SSA and Alt-NHEJ (SA-GFP, 2.8-fold; EJ2-GFP, 2-fold). In contrast, expression of the Trex2-H188A nuclease-deficient mutant caused no statistical difference in such repair (Figure S2B). These results indicate that limiting the persistence of DSBs via Trex2 causes a reduction in Distal-EJ, SSA, and Alt-NHEJ, each of which result in significant deletion mutations.

Next, we analyzed the effect of Trex2 on the HDR reporters shown in Figure 2, using the co-transfection approach described above. From these experiments (Figure 5A and 5B), we found Trex2 expression caused no effect on HDR of DR-GFP, and a minor increase on HDR of DRins-GFP (1.5-fold), where expression of the Trex2-H188A mutant showed no effect on HDR in either reporter (Figure S2B). The increase in HDR for DRins-GFP may be due to Trex2-mediated removal of the I-SceI-overhangs, which would remove some of the mismatched base-pairs between the 5′ DSB end and the template for repair (see Figure S1A). This overhang processing may be particularly important for DRins-GFP, since this reporter may be specifically affected by the mismatched base-pairs between the 5′ end of the DSB and *iGFP*, since the terminus of the 3′ end of the DSB is not homologous to *iGFP* (see Figure 2B). In any case, these results suggest that Trex2 expression does not inhibit HDR, which is distinct from the effects on Distal-EJ, SSA, and Alt-NHEJ.

### Individual genetic factors play distinct roles in repair of DSBs with non-cohesive ends generated by co-expression of I-SceI with Trex2

We next investigated whether repair of DSB ends modified by Trex2 show distinct genetic requirements, focusing on Distal-EJ and HDR. For this, we determined the effect of Trex2 expression on the EJ5-GFP and DR-GFP reporters in each of the DNA repair mutant cell lines described earlier in this study. For each of these cell lines, we first determined whether Trex2 expression promotes I-SceI-resistant EJ products between proximal ends of the 3′ I-SceI site in EJ5-GFP. As described in Figure 4G, this Trex2-mediated EJ product was not detected in *Xrcc4−/−* cells. However, for the other cell lines (*Ercc1−/−*, *Msh2−/−*, *Brca1−/−*, and *Nbs1^n/h^*), we found that Trex2 expression causes the formation of this I-SceI-resistant EJ product to a level that is indistinct from WT (Figure S2C).

**Figure 6:**
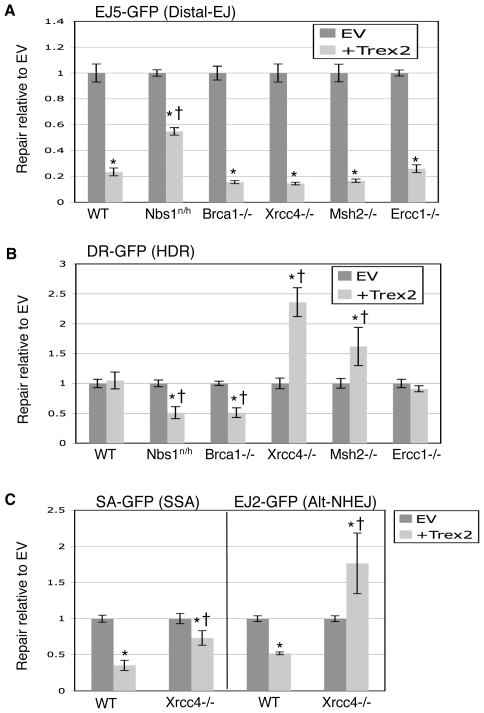
Roles of individual genetic factors during repair of DSBs with non-cohesive ends generated by co-expression of I-SceI with Trex2. Cell lines with individual reporters were co-transfected with expression vectors for I-SceI and Trex2. Repair values are quantified and normalized to parallel co-transfections with I-SceI and EV. (A) Nbs1-deficient cells show a diminished suppression of Distal-EJ from Trex2 expression. Shown are the effects of Trex2 expression on the EJ5-GFP reporter integrated into DNA repair-deficient mouse ES cell lines described in Figure 2 and Figure 3. Asterisks denote a statistical difference from EV (p<0.0001), and the dagger denotes a statistical difference from WT (p<0.0001). (B) Trex2 expression causes a decrease in HDR in cells deficient in Nbs1 and Brca1, but an increase in HDR in cells deficient in Xrcc4 and Msh2. Shown are the effects of Trex2 expression on the DR-GFP reporter in the cell lines shown in A. Asterisks denote a statistical difference from EV (p<0.0001, Msh2 p = 0.001), and the dagger denotes a statistical difference from WT (p<0.0001, Msh2 p = 0.0027). (C) Trex2 expression in *Xrcc4−/−* cells shows an increase in Alt-NHEJ, and a relative increase in SSA compared to WT. Shown are the effects of Trex2 expression on the EJ2-GFP and SA-GFP reporters in *Xrcc4−/−* cells, along with WT. Asterisks and daggers are as in A.

Thus, for each of the cell lines except *Xrcc4−/−*, Trex2 expression promotes the formation of I-SceI-resistant EJ products that are not prone to repeated cutting, which likely limits the persistence of I-SceI-induced DSBs. Subsequently, we quantified the effect of Trex2 expression on the frequency of Distal-EJ and HDR for each of these lines, as determined for WT ES cells in Figure 5A. Beginning with Ercc1, we found that Trex2 expression in *Ercc1−/−* cells affected Distal-EJ and HDR in a manner indistinguishable from WT (Figure 6A and 6B, respectively). In contrast, each of the other cell lines showed distinct effects of Trex2 expression on Distal-EJ and/or HDR.

We found that *Nbs1^n/h^* cells showed a much more mild affect of Trex2 expression on Distal-EJ (1.8-fold compared to 4.2-fold in WT, Figure 6A). Regarding HDR, Trex2 expression in the *Nbs1^n/h^* cells showed a significant decrease in this pathway (2-fold, Figure 6B). In *Brca1−/−* cells, Trex2 caused an inhibition of Distal-EJ that was similar to WT (Figure 6A), but showed a significant decrease in HDR (2-fold, Figure 6B). Thus, with Trex2 expression, which likely results in a less persistent DSB, Nbs1 and Brca1 show an increased role in promoting HDR, and Nbs1 is important for limiting the frequency of Distal-EJ.

In contrast, with *Msh2−/−* cells, we found that Trex2 expression caused an elevation of HDR (1.6-fold, Figure 6B), and a reduction in Distal-EJ that is similar to WT (Figure 6A). In this case, since Trex2-mediated processing of the 3′ I-SceI overhangs may remove a few of the mismatches between *SceGFP* and *iGFP* (see Figure S1A), this result indicates that such processing is particularly important for HDR in *Msh2−/−* cells. As well, since Trex2 expression did not cause an increase in HDR in *Ercc1−/−* cells (Figure 6B), these results further support the notion that Ercc1 and Msh2 play distinct roles during HDR, as described in Figure 2.

Finally, we also addressed how Trex2 expression may affect DSB repair in *Xrcc4−/−* cells. As described above (see Figure 4G), Trex2 expression in these cells does not result in I-SceI-resistant EJ products between proximal ends, suggesting that Xrcc4 is required for EJ of proximal Trex2-modified ends. Regarding distal ends, we found that Trex2 expression caused a decrease in the frequency of Distal-EJ in *Xrcc4−/−* cells (6.9-fold, Figure 6A). These results indicate that Trex2-modified ends are not efficiently repaired by EJ between either proximal or distal ends in the absence of Xrcc4. Accordingly, such products may be more likely to be processed by end resection, and hence be repaired by other pathways. In support of this notion, we find that Trex2 expression caused a substantial increase in HDR and Alt-NHEJ in *Xrcc4−/−* cells (DR-GFP, 2.3-fold; EJ2-GFP, 1.8-fold, Figure 6B and 6C). Furthermore, the suppression of SSA by Trex2 was substantially reduced in *Xrcc4−/−* cells compared to WT (1.3-fold versus 2.8-fold, respectively, Figure 6C). This minor decrease in SSA may reflect a bias towards HDR and/or Alt-NHEJ of Trex2-processed DSB ends in *Xrcc4−/−* cells. In summary, *Xrcc4−/−* cells appear deficient for EJ of Trex2-processed ends, relying more on other repair pathways that likely require end resection, particularly HDR and Alt-NHEJ.

## Discussion

**Figure 7:**
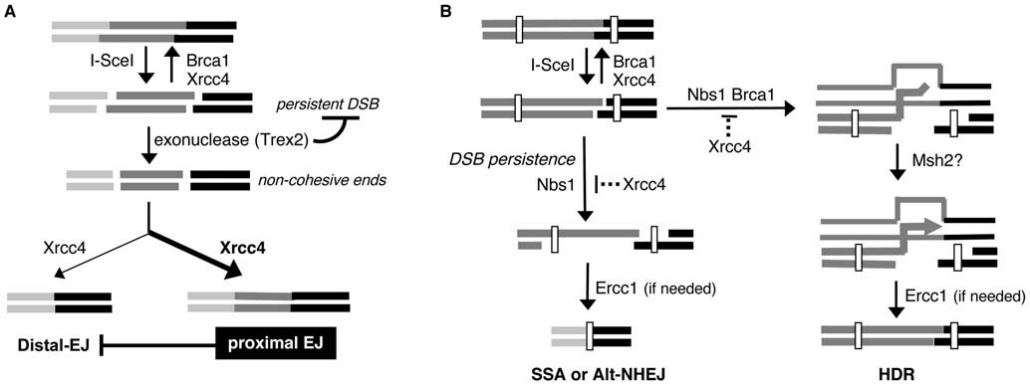
Limiting the persistence of a DSB causes a reduction in repair pathways that result in genetic loss. (A) Proximal EJ is shown to limit Distal-EJ of two tandem DSBs. Xrcc4 and Brca1 are shown to promote EJ of cohesive DSB ends, where repeated cutting by I-SceI results in a persistent DSB. The Trex2 exonuclease is shown as generating non-cohesive DSB ends, which limits the persistence of I-SceI-induced DSBs. Xrcc4 is also shown promoting EJ of non-cohesive ends during both proximal-EJ and Distal-EJ. The bias towards proximal-EJ in WT cells is indicated by the bold arrow (see Figure 5). Similarly, the moderate preference for proximal-EJ via Xrcc4 is indicated by the bold-type. (B) Shown is a model whereby the persistent nature of a DSB is important for SSA and Alt-NHEJ, but not HDR. Nbs1 and Brca1 are shown having an increased role during HDR of a relatively less persistent DSB. End resection that leads to HDR, SSA, and Alt-NHEJ is shown as being suppressed by Xrcc4 and promoted by Nbs1. Msh2 is shown as possibly promoting 3′ end processing prior to nascent DNA synthesis during HDR. Ercc1 is shown to promote the completion of repair pathways if needed, based on the requirement for removal of an extended nonhomologous segment.

Characterizing the cellular conditions that influence the efficiency and fidelity of distinct pathways of chromosome DSB repair provides insight into the process of genome maintenance. One mode of investigation into such pathways has involved monitoring the repair of site-specific chromosomal DSBs, using rare-cutting endonucleases, such as I-SceI. With this approach, we investigated the relative role of individual genetic factors in multiple pathways of repair. Furthermore, we developed a distinct approach for such I-SceI experiments, using expression of Trex2 to promote partial degradation of cohesive I-SceI-induced DSB ends. With this approach, we addressed the role of individual genetic factors during the repair of non-cohesive DSB ends. Moreover, we used the Trex2 approach to limit the persistence of I-SceI-induced DSBs, in that Trex2-mediated processing of DSB ends leads to formation of I-SceI-resistant EJ products, which are not prone to repeated cutting. Using this approach, we provide evidence that limiting the persistence of a DSB can decrease the frequency of repair pathways that lead to genetic loss (Figure 7).

### Roles of individual genetic factors during repair

The findings of the genetic analysis reinforce the notion that some factors are not specific for individual repair pathways per se, but rather may promote a particular mechanistic step that arises during multiple repair events. For example, Msh2, which binds to mismatched bases and promotes their removal during mismatch repair [33], also appears to function during HDR of DSBs, as measured by the DR-GFP and DRins-GFP reporters. Accordingly, Msh2 may be important for removing mismatches between the DSB ends and the template for repair (see Figure S1A). Such mismatch removal may occur during strand exchange and/or prior to strand extension. In support of this notion, expression of Trex2, which could remove the mismatches within the 3′ overhangs (see Figure S1A), promotes HDR in Msh2-deficient cells. Thus, the role of Msh2 during mismatch detection and removal [33] may be important for multiple repair pathways and types of DNA damage.

As another example, we find that Ercc1 promotes repair events that require the removal of an extended nonhomologous insertion, rather than a particular repair pathway. Namely, Ercc1 promotes both SSA, as well as an HDR event that requires removal of a nonhomologous insertion. These results are consistent with the known biochemical function of Ercc1/Xpf in cleaving nonhomologous 3′ ssDNA tails [29], which is also important for nucleotide excision repair [57], EJ of plasmid substrates [30], and gene targeting [58]. So, we suggest that this activity of Ercc1 may be important for the removal of nonhomologous segments during multiple repair pathways.

Similarly, we find that Nbs1 is important for a number of repair events that require access to homology, similar to previous results with the Mre11-complex interacting factor, CtIP [7]. Given that these factors are implicated in ssDNA formation via end resection [9],[11], these results suggest that Nbs1/CtIP-mediated end resection might be a common step among HDR, SSA, and Alt-NHEJ. However, the role of Nbs1 during repair could also reflect functions during DNA end tethering and/or activation of the DNA damage response via ATM [59],[60].

In contrast to Nbs1, we find that Xrcc4 suppresses repair that requires access to homology (HDR, SSA, and Alt-NHEJ). Regarding mechanism, the end-protection function of Xrcc4 [48] may suppress ssDNA formation via end resection, and hence access to homology. As an additional possibility, the EJ functions of Xrcc4-Ligase IV [3] may also be important to effectively compete with repair pathways that require access to homology. In either case, bypass of Xrcc4 function is likely a common mechanistic step during HDR, SSA, and Alt-NHEJ.

Considering the EJ functions of Xrcc4, we find that the non-cohesive ends formed by Trex2 cannot be efficiently repaired by EJ in Xrcc4-deficient cells, using either proximal or distal ends. These findings likely reflect the unique capability of Xrcc4-Ligase IV during ligation of DSB ends that are not stabilized by annealing [3],[56]. As well, this defect in EJ of non-cohesive ends is consistent with the IR hypersensitivity and V(D)J recombination defects of the *Xrcc4−/−* ES cells [47]. Furthermore, we find that Trex2 expression causes an increase in HDR and Alt-NHEJ in *Xrcc4−/−* cells. Thus, Xrcc4-deficient cells show an increased reliance on HDR and Alt-NHEJ for repair of non-cohesive DSB ends. Since HDR and Alt-NHEJ are promoted by CtIP [7], whose functions appear limited to the S/G2/M phases of the cell cycle [9],[61], *Xrcc4−/−* cells would be expected to show an enhanced ability to repair DSBs formed these cell cycle phases. Consistent with this notion, the IR hypersensitivity of Xrcc4-deficient cells is diminished when these cells are exposed to IR in late S phase [62]. In summary, we find that Xrcc4-deficient cells show defects in EJ repair of non-cohesive DSB ends, as well as an increased reliance on HDR and Alt-NHEJ for repair of such DSB ends.

Finally, while Brca1 is similar to Nbs1 in promoting HDR and SSA [8], we also found that Brca1 supports S+DEJ and suppresses the total frequency of Distal-EJ, which suggests that Brca1 could be important for I-SceI-restoration EJ. In contrast, Brca1 is not important for EJ repair of Trex2-processed ends, which lack significant microhomology. These findings raise the possibility that Brca1 may be particularly important for EJ of cohesive ends that do not require end resection. Consistent with this notion, previous studies have shown a role for Brca1 during EJ repair of plasmid substrates with cohesive ends [51],[52]. Thus, the function of Brca1 during repair cannot be limited to promoting access to homology via ssDNA formation [11],[63], which is also supported by findings that Brca1 may associate with a number of multi-subunit complexes [64], and includes additional functions apart from E3 ligase activity [65]. In summary, with this genetic analysis, we have provided some distinct findings on the role of individual factors during repair of both cohesive and non-cohesive DSB ends.

### Limiting the persistence of a DSB reduces the frequency of Distal-EJ

We addressed how the persistence of a DSB affects the frequency of mutagenic repair events. For this, we used expression of Trex2, which we find promotes the formation of I-SceI-resistant EJ products, which we suggest limits the persistent nature of I-SceI-induced DSBs. While Trex2 is likely promoting these products directly through its exonuclease function, it is certainly possible that Trex2 could additionally be recruiting other factors to facilitate the formation of I-SceI-resistant EJ products. In either case, with this approach, we have found that limiting the persistence of a DSB reduces the frequency of deletion mutations caused by Distal-EJ, SSA, and Alt-NHEJ.

Regarding the effect on Distal-EJ, this result suggests that the relative persistence of DSBs can affect the fidelity of end-pairing during EJ. Persistent breaks could lead to a failure of proximal end-pairing by a number of mechanisms, depending on which factors perform this pairing function. As one example, the DNA tethering activity of the Mre11-complex [59],[60] may support proximal end-pairing during EJ [21]. In this model, persistent DSBs could signal a direct disruption of the Mre11-complex tethering activity, which could lead to the loss of proximal end-pairing. Alternatively, the Mre11-complex may not be able to sustain correct end pairing under the conditions of a persistent DSB.

Consistent with such models, we find that Nbs1 has no effect on Distal-EJ of relatively persistent DSBs (I-SceI expression alone). In contrast, we find that Nbs1 is important to inhibit Distal-EJ of relatively less persistent DSBs (expression of both I-SceI and Trex2). Thus, Nbs1 may promote correct end-pairing during EJ, but in a manner that is less efficient for persistent DSBs. In contrast to Nbs1, Xrcc4 and Brca1 are important for inhibition of Distal-EJ of persistent DSBs (I-SceI expression alone, see Figure 3D, Figure S3E). In summary, we suggest that DSB persistence may affect the relative roles of factors and complexes involved in end-pairing during EJ, where the Trex2 approach described here may facilitate future investigation into this process.

### The influence of Trex2 and DSB persistence on repair requiring access to homology

In addition to affecting Distal-EJ, expression of Trex2 also caused a significant inhibition of SSA and Alt-NHEJ, but not HDR. Considering one model, the Trex2 protein may directly inhibit end resection, perhaps by blocking access of a resection factor to the DSB. However, such direct inhibition does not explain the differential effect of Trex2 on SSA and Alt-NHEJ versus HDR. As well, this model is inconsistent with the findings that Trex2-H188A does not affect repair, as this protein lacks the exonuclease activity, but shows only a 60% reduction in DNA binding activity [54].

Perhaps more likely, Trex2 expression limits the persistence of I-SceI-induced DSBs, which decreases the probability that end resection will be initiated, but in a manner that diminishes Alt-NHEJ and SSA, but not HDR. This differential effect between the pathways may be related to the unique requirement for the sister chromatid during HDR, which is the preferred template even if an intrachromosomal repeat is present [66]. Thus, considering this model, one of the earliest mechanistic steps following a DSB could be attempts to detect the presence of the sister chromatid. If the sister chromatid is found, this event could trigger Xrcc4-bypass and promotion of end resection via CtIP and the Mre11-complex [9],[11]. Given the presence of the sister chromatid, such end resection would likely be followed by efficient strand invasion and HDR. This model is supported by our findings that factors implicated in end resection, Nbs1 and Brca1 [11],[63], show an elevated importance for HDR of a less persistent DSB (i.e. when Trex2 is expressed). Although, this result could also reflect a role for Nbs1 during direct detection of the sister chromatid, given the DNA tethering capabilities of the Mre11-complex [59],[60]. To summarize this model, sister chromatid detection would precede EJ to trigger end resection, such that the persistent nature of a DSB may not be particularly relevant for the frequency of HDR.

Furthermore, in considering this model, we note that the persistence of a DSB has been shown to differentially affect HDR versus SSA in another set of findings. Specifically, a previous study compared repair of both I-SceI-generated DSBs and IR-induced DSBs, where the I-SceI DSBs would be expected to be more persistent than IR DSBs [67]. In this study, I-SceI-generated DSBs were found to stimulate both Rad51-dependent and Rad51-independent repair pathways, which are measures of HDR and SSA, respectively. In contrast, less persistent IR DSBs showed a strong preference for Rad51-dependent repair (HDR). Thus, this previous study is consistent with the notion that the persistence of I-SceI-generated DSBs may be more important for SSA than HDR.

As well, it is notable that HDR of the DRins-GFP reporter is also not inhibited by Trex2 expression. This reporter is similar to DR-GFP in that it requires strand invasion with a homologous template, but is similar to SSA in that it requires Ercc1-dependent removal of an insertion. Thus, the Trex2/DRins-GFP result further supports the notion that strand invasion may be the mechanistic step of HDR that is relatively unaffected by the persistence of a DSB. Regarding another consideration with this reporter, the finding that HDR is less efficient for DRins-GFP than DR-GFP may suggest that limiting efficient strand invasion to one end of the DSB may suppress HDR. These data raise the possibility that strand invasion of both DSB ends may be required for efficient HDR, which is evocative of the classical double-strand break repair model [68].

Finally, since a number of investigators have been developing meganucleases to initiate gene targeting [69], we suggest that co-expression of such meganucleases with Trex2 may provide a means to maintain efficient homologous targeting by HDR, while simultaneously suppressing repair events that are genome destabilizing. In general, we suggest that co-expression studies of meganucleases with Trex2 will lead to additional insight into the pathways that support genome maintenance.

## Materials and Methods

### Plasmids and cell lines

The DRins-GFP reporter is a derivative of pim-DR-GFP#6 [70], where a 464 nt. BglII/AvrII intronic fragment of the mouse *Rb* gene [24] was cloned downstream of the I-SceI site. Complementation/expression cassettes for each gene were cloned into pCAGGS-BSKX [12]. The ERCC1 and Nbs1 complementation vectors have been described previously [8],[42], the Msh2 insert was derived from pHA801 [34], the XRCC4 insert was derived from clone GI:16740906, ATCC#10659357. The mouse *Trex2* coding sequence is present within a single exon [22], and thus was generated from PCR amplification of mouse ES genomic DNA for cloning into pCAGGS-BSKX [12], using these primer sequences: 5′cagctctaggcctcattgtt and 5′agagcctggatgaatggatg. The expression vector of the Trex2-H188A mutant was generated by site-directed mutagenesis of the above expression vector with the Quikchange method (Stratagene) using the primer 5′gctgaacccagtgctgccgcttcagcagaaggtgatgtgc along with the complementary primer.

Chromosomal integration of reporters into mouse ES cells was performed by electroporation using a pulse of 700–730 V 10 µF. Electroporation cuvettes contained 10^7^ cells in 0.8 ml of Optimem (Invitrogen), along with 20–30 µg of linearized plasmid for random integration and 70 µg of linearized plasmid for *Pim1* targeting. Culturing of mouse ES cells on gelatin, and targeting of reporters to the *Pim1* locus was performed as previously described [7]. The reporters targeted to *Pim1* are DR-GFP, DRins-GFP, EJ2-GFP, EJ5-GFP into WT and *Xrcc4−/−*, and EJ5-GFP into *Trex2^null^* and *Brca1−/−*. Otherwise, individual reporters were introduced by random integration using the linked *puro* gene by selecting for clones in 1–2 µg/ml puromycin, where an intact copy of the reporter was confirmed by Southern blotting, as described previously [7],[26].

### Repair assays

To measure repair, 10^5^ cells were plated onto 12 well plates, and transfected the next day with 3.6 µl of Lipofectamine 2000 (Invitrogen) mixed with 0.8 µg of pCBASce, along with 0.4 µg of either empty vector (pCAGGS-BSKX), or the relevant complementation/expression vector. Transfection was performed in 1 ml of antibiotic-free media for 4 hours, after which the transfection media was replaced with regular media. The percentage of GFP positive cells was quantified by flow cytometric analysis (FACS) 3 d after transfection on a Cyan ADP (Dako), from cells suspended and fixed in phosphate-buffered formaldehyde. Amplification of PCR products from sorted GFP+ cells, associated restriction digests, and quantification of bands were performed as previously described [7],[15], where KNDRF and KNDRR are shown as p3 and p2 respectively, primer p1 is EJ5purF: 5′agcggatcgaaattgatgat, primer p4 is EJ5purR: 5′ cttttgaagcgtgcagaatg, and DRins-GFP amplifications use KNDRF and DRRT6: 5′aggttcagggggaggtgt.

To ensure complete I-SceI digestion, PCR products were purified using a GFX column (GE), and digested for 1 h (37°C) with 5 U of I-SceI (NEB), followed by an adding another 5 U of I-SceI and 1 h of digestion. With this protocol, we always ensure complete cutting with a control PCR template that contains an intact I-SceI site (see Figure 4A), and further ensure that our experimental samples contain less or equal amounts of PCR product as these controls, to avoid any possibility of problems with excess substrate affecting complete cutting [45].

Repair frequencies are the mean of a minimum of four transfections where error bars represent the standard deviation from the mean. In most cases, repair frequencies are shown relative to samples co-transfected with I-SceI and an empty vector (EV). For this calculation of fold-complementation, the percentage of GFP+ cells from each sample was divided by the mean value of the EV samples treated in the parallel experiment. Statistical analysis was performed using the unpaired *t*-test.

### Immunoblot analysis

Transfections were performed as in the repair assays, and 2 d after transfection, protein was isolated by repeated freeze/thawing in NETN buffer (20 mM Tris pH 8, 100 mM NaCl, 1 mM EDTA, 0.5% IGEPAL, 1 mM DTT) with Protease Inhibitor Cocktail (Roche). Protein was separated on 4–12% SDS-PAGE, and probed with anti-NBS1 antibody (Bethyl labs, A301-284A) and HRP-conjugated anti-rabbit (Santa Cruz Biotechnology, sc-2004), or probed with HRP-conjugated anti-GAPDH (Abcam, ab9482), and developed with ECL (GE).

## Supporting Information

Figure S1 A diagram for DR-GFP/Trex2 and additional controls for the complementation experiments. (A) Shown is the divergence between *SceGFP* and *iGFP* gene segments in the DR-GFP reporter at the position of the I-SceI cut site, along with predicted changes in this divergence following Trex2-mediated degradation of the I-SceI overhangs. Trex2 is shown as completely degrading the entire I-SceI overhang, which need not be the case. (B) Complementation vectors for Msh2, XRCC4, and ERCC1 express the predicted protein. Co-tranfections of I-SceI and EV or the relevant complementation vector was performed in the relevant mutant cell line with identical conditions as the repair assays, along with parallel transfections of EV in WT ES cells. Following 48 h after transfection, protein extraction and immunoblotting were performed as described for Nbs1 in the Materials and Methods. Shown are immunoblot signals from these transfections for ERCC1 (Ab1: SCBT sc-10785, Ab2: SCBT sc-17809), Msh2 (Abcam ab16833), XRCC4 (SCBT sc-8285), and GAPDH (Abcam ab9482). Ercc1 immunoblotting signal is not detected in WT ES cells, as described in the original report with the *Ercc1−/−* cell line [31]. Accordingly, these cells were complemented with an expression vector for human ERCC1, as this protein can be detected by immunoblotting [31]. We have used the same complementation approach with human ERCC1, and show immunoblotting signals from two different antibodies for illustration. (C) Quantification of S+DEJ of sorted GFP+ cells. Shown is the mean I-SceI restoration (S+DEJ) from amplification products from GFP+ sorted samples as shown in Figure 3, calculated relative to samples from WT ES cells.
(0.32 MB TIF)

Figure S2 Additional controls for the Trex2 experiments. (A) Transfection of Trex2 does not appear to cause elevated γH2AX, a marker for chromosome breaks. Transfections of EV and Trex2 were performed in WT ES cells as described in Figure 4. Following 48 h after transfection, cells were incubated with NETN as described in the Materials and Methods, and subsequently histones were extracted with 0.2 M HCl, and analyzed with 12% SDS-PAGE and immunoblotting. Shown are immunoblot signals from γH2AX (Cell Signaling #2577), as well as ponceau-S signals of histone H3 from the identical blot. (B) Expression of a nuclease-deficient mutant of Trex2 (Trex2-H188A) showed no effect on repair in WT ES cells. WT ES cells with individual reporters were transfected with I-SceI along with an expression vector for Trex2-H188A or EV. Repair values are quantified and normalized to the parallel EV transfections, as in Figure 5A. (C) Co-expression of I-SceI and Trex2 in WT, *Ercc1−/−*, *Msh2−/−*, *Nbs1^n/h^*, and *Brca1−/−* cells causes efficient formation of I-SceI-resistant EJ products. Co-transfections of I-SceI with either EV or Trex2, and subsequent analysis of I-SceI-resistant EJ products at the 3′ I-SceI site of EJ5-GFP, were performed as described for WT in Figure 4B. Shown is the mean percentage of I-SceI-resistant EJ products from at least three independent transfections for each cell line.
(0.19 MB TIF)

Figure S3 Primary repair data. Repair levels for each reporter are shown with each cell line, to allow comparison across cell lines. Shown are repair levels for (A) DR-GFP (HDR), (B) DRins-GFP (insHDR), (C) SA-GFP (SSA), (D) EJ2-GFP (Alt-NHEJ), and (E) EJ5-GFP (Distal-EJ). As noted, +comp refers to the transfection of the relevant complementation vector for each mutant line. The error bars are somewhat larger in the primary repair data, as we observe greater experimental variation in the absolute levels of repair, as compared to the consistent fold-effect of complementation on repair (see Figure 2 and Figure 3). Asterisks denote a statistical difference between +comp and EV (DR-GFP, p<0.0001; DRins-GFP, p<0.01; SA-GFP, p<0.008; EJ2-GFP, p<0.007). For EJ5-GFP, the dagger denotes a statistical difference from WT (p<0.0001). The double-dagger indicates cell lines that show a consistent statistical difference when +comp values are compared to parallel EV transfections (see Figure 2 and Figure 3), but where a statistically significant difference is not observed in the mean of the primary repair data; due again to the experimental variation in absolute levels of repair versus the relatively consistent fold-effect of complementation.
(0.38 MB TIF)
