# Supplementary figures and images for: Limiting the Persistence of a Chromosome Break Diminishes Its Mutagenic Potential
Source: PLoS Genet. 2009 Oct 16;5(10):e1000683. doi: 10.1371/journal.pgen.1000683 (PMC2752804; doi:10.1371/journal.pgen.1000683)

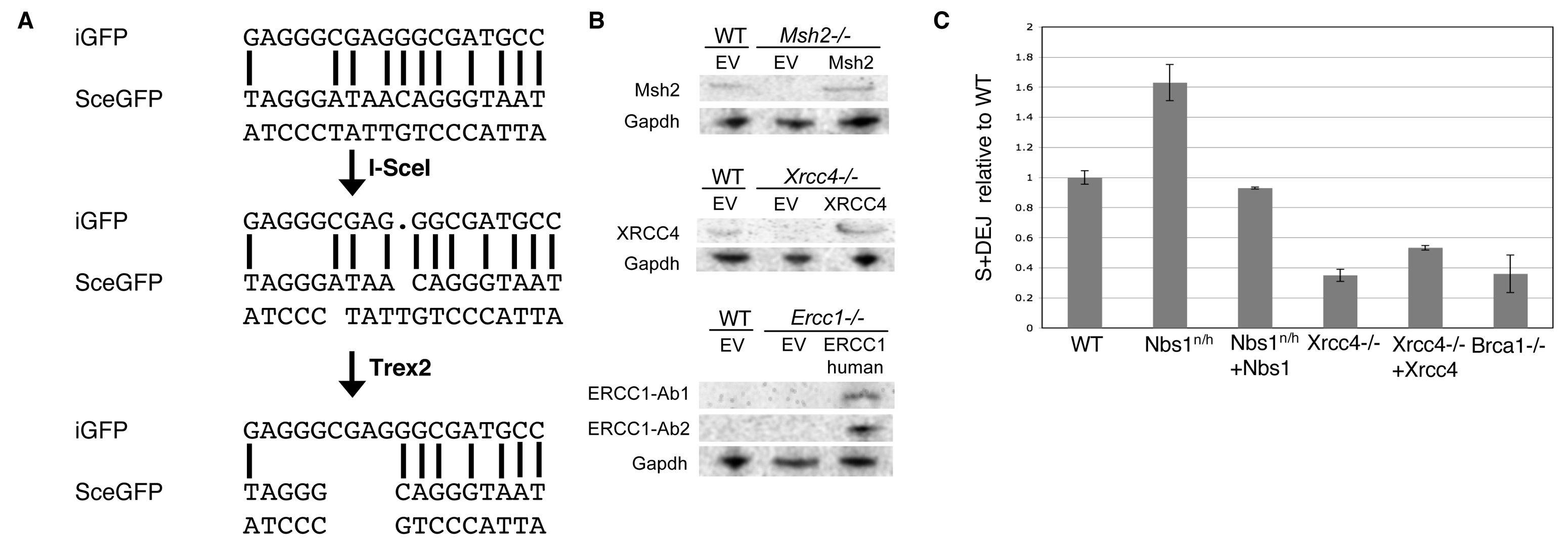

Supplement: Figure S1 — A diagram for DR-GFP/Trex2 and additional controls for the complementation experiments. (A) Shown is the divergence between SceGFP and iGFP gene segments in the DR-GFP reporter at the position of the I-SceI cut site, along with predicted changes in this divergence following Trex2-mediated degradation of the I-SceI overhangs. Trex2 is shown as completely degrading the entire I-SceI overhang, which need not be the case. (B) Complementation vectors for Msh2, XRCC4, and ERCC1 express the predicted protein. Co-tranfections of I-SceI and EV or the relevant complementation vector was performed in the relevant mutant cell line with identical conditions as the repair assays, along with parallel transfections of EV in WT ES cells. Following 48 h after transfection, protein extraction and immunoblotting were performed as described for Nbs1 in the Materials and Methods. Shown are immunoblot signals from these transfections for ERCC1 (Ab1: SCBT sc-10785, Ab2: SCBT sc-17809), Msh2 (Abcam ab16833), XRCC4 (SCBT sc-8285), and GAPDH (Abcam ab9482). Ercc1 immunoblotting signal is not detected in WT ES cells, as described in the original report with the Ercc1−/− cell line [31]. Accordingly, these cells were complemented with an expression vector for human ERCC1, as this protein can be detected by immunoblotting [31]. We have used the same complementation approach with human ERCC1, and show immunoblotting signals from two different antibodies for illustration. (C) Quantification of S+DEJ of sorted GFP+ cells. Shown is the mean I-SceI restoration (S+DEJ) from amplification products from GFP+ sorted samples as shown in Figure 3, calculated relative to samples from WT ES cells. (0.32 MB TIF) [file pgen.1000683.s001.tif]

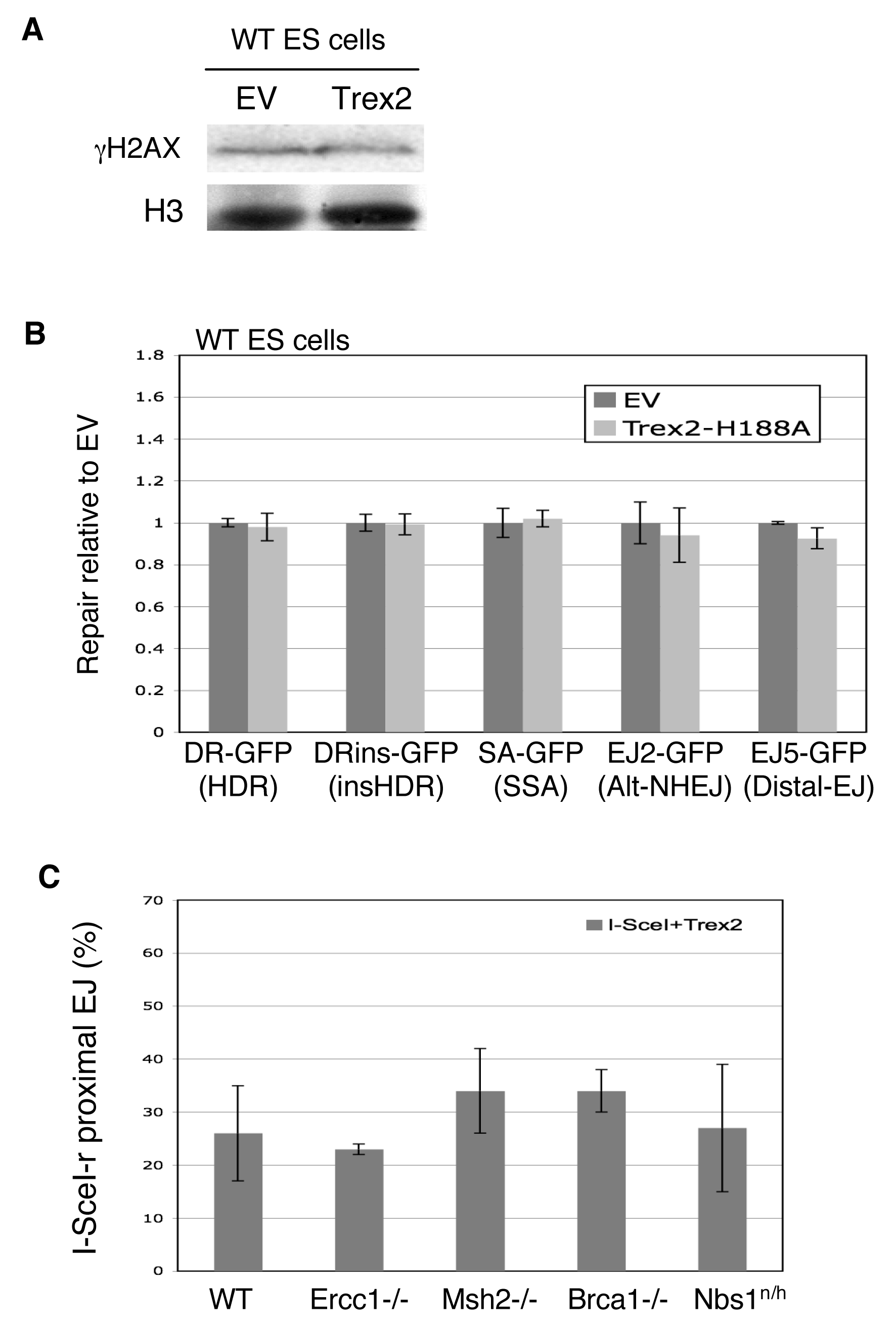

Supplement: Figure S2 — Additional controls for the Trex2 experiments. (A) Transfection of Trex2 does not appear to cause elevated γH2AX, a marker for chromosome breaks. Transfections of EV and Trex2 were performed in WT ES cells as described in Figure 4. Following 48 h after transfection, cells were incubated with NETN as described in the Materials and Methods, and subsequently histones were extracted with 0.2 M HCl, and analyzed with 12% SDS-PAGE and immunoblotting. Shown are immunoblot signals from γH2AX (Cell Signaling #2577), as well as ponceau-S signals of histone H3 from the identical blot. (B) Expression of a nuclease-deficient mutant of Trex2 (Trex2-H188A) showed no effect on repair in WT ES cells. WT ES cells with individual reporters were transfected with I-SceI along with an expression vector for Trex2-H188A or EV. Repair values are quantified and normalized to the parallel EV transfections, as in Figure 5A. (C) Co-expression of I-SceI and Trex2 in WT, Ercc1−/−, Msh2−/−, Nbs1n/h, and Brca1−/− cells causes efficient formation of I-SceI-resistant EJ products. Co-transfections of I-SceI with either EV or Trex2, and subsequent analysis of I-SceI-resistant EJ products at the 3′ I-SceI site of EJ5-GFP, were performed as described for WT in Figure 4B. Shown is the mean percentage of I-SceI-resistant EJ products from at least three independent transfections for each cell line. (0.19 MB TIF) [file pgen.1000683.s002.tif]

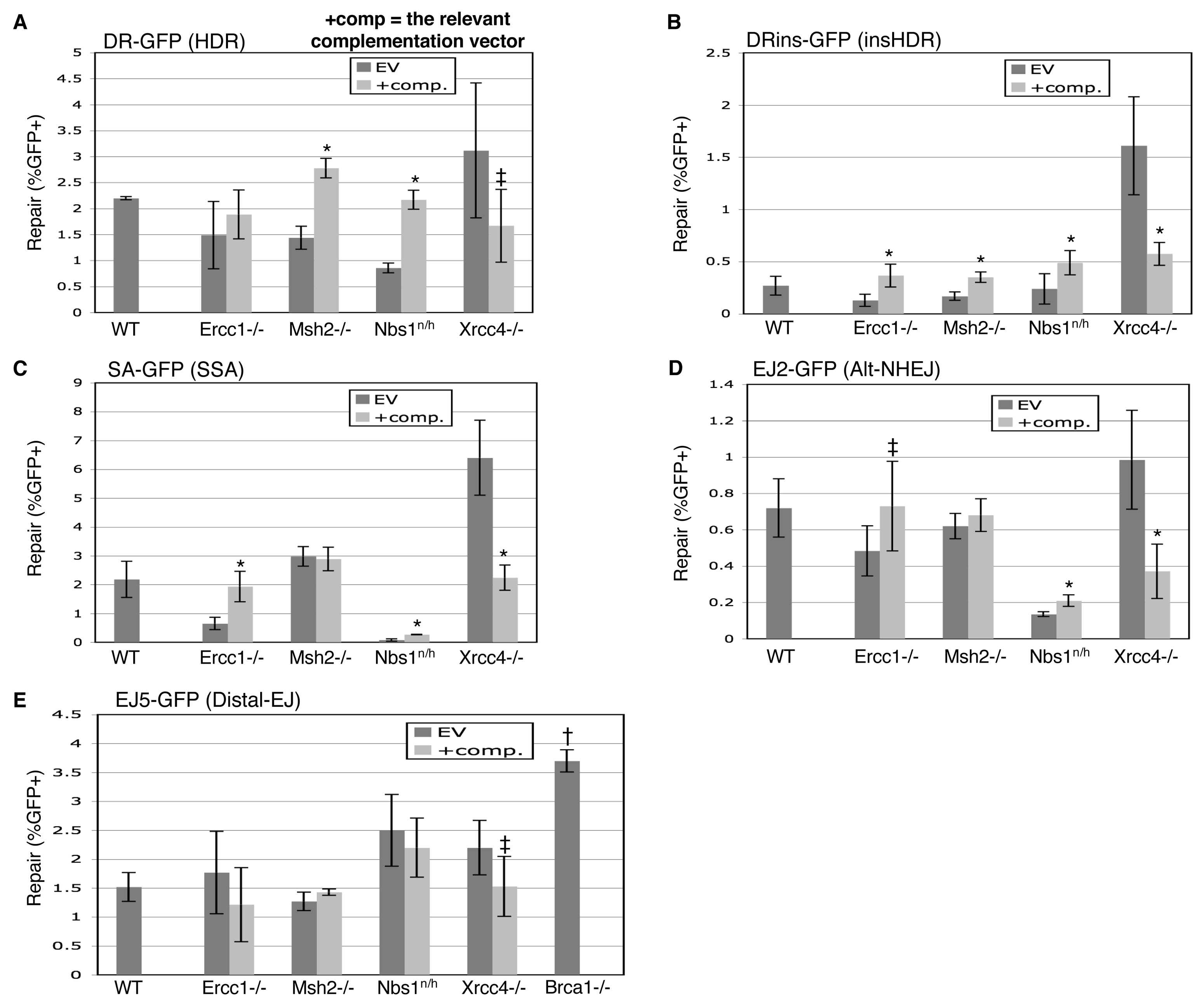

Supplement: Figure S3 — Primary repair data. Repair levels for each reporter are shown with each cell line, to allow comparison across cell lines. Shown are repair levels for (A) DR-GFP (HDR), (B) DRins-GFP (insHDR), (C) SA-GFP (SSA), (D) EJ2-GFP (Alt-NHEJ), and (E) EJ5-GFP (Distal-EJ). As noted, +comp refers to the transfection of the relevant complementation vector for each mutant line. The error bars are somewhat larger in the primary repair data, as we observe greater experimental variation in the absolute levels of repair, as compared to the consistent fold-effect of complementation on repair (see Figure 2 and Figure 3). Asterisks denote a statistical difference between +comp and EV (DR-GFP, p<0.0001; DRins-GFP, p<0.01; SA-GFP, p<0.008; EJ2-GFP, p<0.007). For EJ5-GFP, the dagger denotes a statistical difference from WT (p<0.0001). The double-dagger indicates cell lines that show a consistent statistical difference when +comp values are compared to parallel EV transfections (see Figure 2 and Figure 3), but where a statistically significant difference is not observed in the mean of the primary repair data; due again to the experimental variation in absolute levels of repair versus the relatively consistent fold-effect of complementation. (0.38 MB TIF) [file pgen.1000683.s003.tif]
